# National Poison Center Trends in GLP-1 Receptor Agonist Exposures Following FDA Approval for Weight Loss

**DOI:** 10.1007/s13181-026-01121-z

**Published:** 2026-02-03

**Authors:** Jordan Miller, Robert Miller, Shawn M. Varney, David Han

**Affiliations:** 1 College of AI, Cyber and Computing, UT San Antonio, San Antonio, TX USA; 2 South Texas Poison Center, Department of Emergency Medicine, Long School of Medicine, UT San Antonio, San Antonio, TX USA

**Keywords:** Glucagon-like peptide receptor agonists, Semaglutide, Poison centers, Pharmacovigilance

## Abstract

**Introduction:**

Glucagon-like peptide-1 receptor agonists (GLP-1 RAs) are therapies for type 2 diabetes whose use expanded sharply after semaglutide’s 2021 approval for obesity. Although gastrointestinal effects are well described, national patterns of acute GLP-1 RA exposures are poorly characterized. This study evaluated trends in GLP-1 RA exposures reported to U.S. poison centers, focusing on demographic shifts, exposure circumstances, and clinical outcomes before and after the 2021 FDA approval.

**Methods:**

We analyzed human GLP-1 RA exposures reported to the National Poison Data System from 2012 to 2023, using July 1, 2021, to define pre- and post-approval periods. Demographics, exposure characteristics, therapies, and medical outcomes were compared using standardized statistical tests. Quarterly call counts were modeled with segmented Poisson regression to assess changes in reporting trajectory.

**Results:**

A total of 10,033 exposures were identified (3,113 pre-approval; 6,920 post-approval). Semaglutide predominated post-approval (64.2%). The exposed population shifted younger and more female. Most cases were unintentional therapeutic errors with mild gastrointestinal symptoms. The proportion managed in or referred to a health care facility increased from 23.0% to 33.5% (RR = 1.46, [95% CI: 1.36, 1.57], *p* < 0.001). Segmented Poisson modeling demonstrated a significant inflection in call volume, with semaglutide exposures increasing an additional 9.9% per quarter after approval.

**Conclusions:**

GLP-1 RA exposures rose sharply following semaglutide’s weight-loss approval, accompanied by increased health care utilization despite generally mild clinical effects. Although multiple factors likely contributed to these trends, improved patient counseling and clearer poison center guidance may help reduce preventable therapeutic errors and unnecessary emergency evaluation.

**Supplementary Information:**

The online version contains supplementary material available at 10.1007/s13181-026-01121-z.

## Introduction

Glucagon-like peptide-1 receptor agonists (GLP-1 RAs) have been used in the treatment of type 2 diabetes since the 2005 approval of exenatide, the first agent in this class. These agents mimic the endogenous hormone GLP-1, enhancing glucose-dependent insulin secretion, suppressing glucagon, delaying gastric emptying, and reducing food intake [1]. Early trials showed that twice-daily subcutaneous exenatide improved glycemic control and produced sustained weight loss in patients with type 2 diabetes [2–4], and similar effects were later observed in obese individuals without diabetes [5]. Liraglutide, a once-daily subcutaneous GLP-1 RA, also improved glycemic parameters and reduced body weight in both diabetic and non-diabetic populations [6, 7]. These findings supported the 2014 approval by the U.S. Food and Drug Administration (FDA) of liraglutide for chronic weight management in patients aged 12 years and older with obesity and at least one weight-related comorbidity [8].

Semaglutide, a once-weekly subcutaneous formulation, demonstrated greater reductions in hemoglobin A1c and body weight compared with placebo and several active comparators in the SUSTAIN trials [9]. The STEP 1 study later demonstrated a 12.4% mean reduction in body weight from baseline to week 68 with 2.4 mg semaglutide [10], leading to FDA approval for use in adults with obesity [11]. The combination of significant weight loss efficacy, weekly dosing convenience, and expanding clinical adoption has coincided with a substantial rise in GLP-1 RA prescribing across U.S. subpopulations, with marked increases observed between 2020 and 2024, particularly among individuals with obesity or concomitant diabetes [12].

GLP-1 agonists promote weight loss by slowing the rate at which food is emptied from the stomach, which can reduce appetite and promote satiety. This can lead to decreased food intake and result in favorable changes in body weight and composition, including reductions in body fat mass and improvements in insulin sensitivity [13, 14]. Emerging clinical observations suggest that some patients with overlapping obesity and addiction dysregulation may experience benefits in both domains, although the mechanisms remain uncertain [15].

Delayed gastric emptying contributes to hallmark side effects such as nausea and vomiting, which can be severe, persist for several days, and lead to dehydration and electrolyte abnormalities [13, 14]. The relationships between GLP-1 RAs and pancreatitis and, separately, pancreatic cancer remain inconclusive, with retrospective analyses of clinical trial data and observational studies producing inconsistent results; some studies suggest increased risk while others show no change [16–19]. Similarly, an elevated risk of hypoglycemia has been reported, particularly in patients already at risk, such as those prescribed insulin or sulfonylureas [20]. However, some evidence suggests that this hypoglycemia may be specific to individual GLP-1 RAs and not a class effect [21].

The widespread interest in GLP-1 receptor agonists for weight loss, particularly amid drug shortages, has renewed clinical and regulatory attention to their safety profile, yet the clinical profile of acute exposures remains poorly understood. Reports of adverse effects following acute or acute-on-chronic exposures remain incompletely characterized, and existing literature has not clarified whether new toxicity patterns emerge when these drugs are used primarily for obesity rather than diabetes.

Beyond clinical effects, characterizing temporal trends is necessary to contextualize the rise in GLP-1 RA exposures. While simple pre/post comparisons can demonstrate that exposures have increased, such approaches do not account for the strong time-dependent nature of poison center data. Call volumes often exhibit non-linear growth and seasonal fluctuations, which can obscure true inflection points if not modeled properly. Statistical modeling allowed us to formally test whether the increase in GLP-1 RA exposures after the FDA’s approval of semaglutide in 2021 represents a significant change in trajectory beyond what would be expected from underlying prescribing growth. This approach also supports sensitivity analyses using alternative specifications (e.g., Poisson, negative binomial), providing a robust characterization of temporal patterns.

### Objectives

This study had two objectives. First, we sought to clinically characterize GLP-1 RA exposures, including their frequency, demographics, and adverse effects reported to the National Poison Data System (NPDS), with a focus on changes following the FDA’s approval of semaglutide for chronic weight management. Specifically, we hypothesized that the post-approval period would be associated with a significant increase in exposures and healthcare facility utilization driven by a rise in therapeutic errors. We based this hypothesis on historical data identifying device-related error rates of 17–57% for the first dose of exenatide, liraglutide, and lixisenatide pens, compared with 17–43% for the second dose; given the weight loss approval, we anticipated a substantial increase in first-time use [22]. This expectation is further supported by recent single-center studies demonstrating that therapeutic errors account for approximately 69% [23] to 90% [24] of reported GLP-1 RA exposures. Second, from a statistical standpoint, our objective was to model the temporal trends in call frequency to identify the best-fitting model that describes the observed increase in reports. This analysis aimed to evaluate whether poison center data can reliably detect drug-related inflection points in national exposure trends over time.

## Methods

We conducted a retrospective analysis of human exposures to GLP-1 RAs reported to the National Poison Data System (NPDS) from January 1, 2012 through December 31, 2023. Maintained by America’s Poison Centers, the NPDS aggregates de-identified case records from all regional poison centers across the United States and its territories. Case data are coded in near real-time by Specialists in Poison Information, registered nurses and pharmacists with specialized training in clinical toxicology, during telephone consultations with the public and healthcare professionals.

Exposures were included if the primary substance was a GLP-1 RA (exenatide, liraglutide, dulaglutide, albiglutide, semaglutide, tirzepatide, or an unspecified GLP-1 RA). Animal exposures and information-only calls were excluded. The FDA approved once-weekly semaglutide for weight loss on June 4, 2021 [25]. Exposures occurring on July 1 were included in the post-approval group. This cut-point aligns with the start of the standard calendar quarter and accounts for the expected short latency between regulatory approval and the medication’s diffusion into routine clinical use.

Case-level data included demographics (age, sex), exposure circumstances, chronicity (acute, acute-on-chronic, or chronic), exposure site, caller site, management site, level of health care facility (HCF) care, medical outcome, clinical effect duration, clinical effects, and therapies. Patient weight was excluded from the analysis due to a high proportion of missing data (77.7%) and data quality concerns, such as implausible values, and inconsistent units. Similarly, quantitative dosing data were excluded from the analysis due to significant heterogeneity in reporting. Reports frequently conflated volume (mL) with strength (mg), used non-standard units such as “clicks” of a pen device or “units” on an insulin syringe, and often involved compounded formulations with variable or unknown concentrations, making standardization of dose and meaningful quantification impossible.

Clinical effects in NPDS are captured as individual binary indicators coded as “related,” “not related,” or “unknown if related,” and these variables were used to calculate frequencies and percentages for each period. For the primary analysis, symptoms coded as “unknown if related” were grouped with those coded as “related.” This approach prioritized sensitivity by ensuring that rare but clinically meaningful effects were not excluded solely because the initial attribution was equivocal. To assess the impact of this decision, a sensitivity analysis compared the proportion of symptoms coded as “unknown” between the pre- and post-approval periods. Therapy variables, coded as “recommended,” “performed,” “recommended and performed,” or “not recommended/performed,” were collapsed into aggregate summary frequencies for analysis.

This study was determined to be exempt from institutional review board oversight because it involved secondary analysis of de-identified poison center surveillance data. Although the analysis was primarily descriptive, reporting followed the Strengthening the Reporting of Observational Studies in Epidemiology (STROBE) framework in all domains where it was applicable.

### Statistical Analysis

We began the analysis by defining the comparison groups and assessing demographic differences between periods. Welch’s t-tests were used to compare mean age, Wilcoxon rank-sum tests were used to compare medians when distributions were skewed, and chi-square or Fisher’s exact tests were used for comparisons of proportions depending on expected cell sizes. The primary outcome for assessing healthcare utilization was the proportion of exposures resulting in transfer or referral to a health care facility (HCF). Comparisons between periods were conducted using chi-square tests, and effect sizes were quantified with risk ratios and 95% confidence intervals.

To distinguish new post-approval exposures from the continuation of pre-existing patterns, we conducted an exploratory procedure to construct an adjusted post-approval age distribution. This descriptive approach, intended to approximate the characteristics of the emergent weight-loss population, involved subtracting pre-approval frequencies from post-approval counts at each age; negative values were truncated at zero. Prior to comparing means, the distribution of patient age was formally assessed and found to be significantly skewed (non-normal), which is expected in spontaneous reporting data. Although Welch’s t-test is robust in large samples, Wilcoxon rank-sum tests were also performed to provide a conservative, non-parametric confirmation of the shift in central tendency.

For the temporal analysis of call frequency, quarterly call counts were modeled using segmented Poisson regression to evaluate changes in level and slope following the July 2021 approval date. This method was selected because segmented regression is well suited to testing whether a known event corresponds to a statistically significant inflection in reporting trajectory beyond background growth, and because difference-in-difference models require a parallel control group not available in NPDS data. Polynomial regression models (orders one through four) were fit solely for exploratory visualization and were not used for inference. Because overdispersion was present in exploratory models, both Poisson and negative binomial specifications were fit; final inference relied on the Poisson model, which demonstrated superior fit as assessed by Akaike Information Criterion (AIC) and pseudo-R² statistics. Variable selection methods, including least absolute shrinkage and selection operator (LASSO), elastic net, and stepwise selection, were used in an exploratory capacity to assess predictor stability across model specifications. Model performance was evaluated by comparing observed and predicted call frequencies and by visual inspection of residuals. All analyses were two-tailed with α = 0.05 and were conducted in R (version 2025.05.1 Build 513), with outputs exported to Excel or image formats for integration into the manuscript.

## Results

A total of 10,033 GLP-1 RA exposures were reported, comprising *n* = 3,113 before July 1, 2021, and *n* = 6,920 on and after this date. The post-approval period therefore showed a marked increase in reporting volume, with 2.2 times more exposures than the pre-approval period. Our segmented time-series analysis confirmed that the observed increase in exposures represented a significant change in the trend in reporting volume. The incidence rate of semaglutide calls accelerated by an additional 9.9% per quarter after the July 2021 approval (IRR = 1.10 [95% CI: 1.08,1.11], *p* < 0.001).

**Fig. 1:**
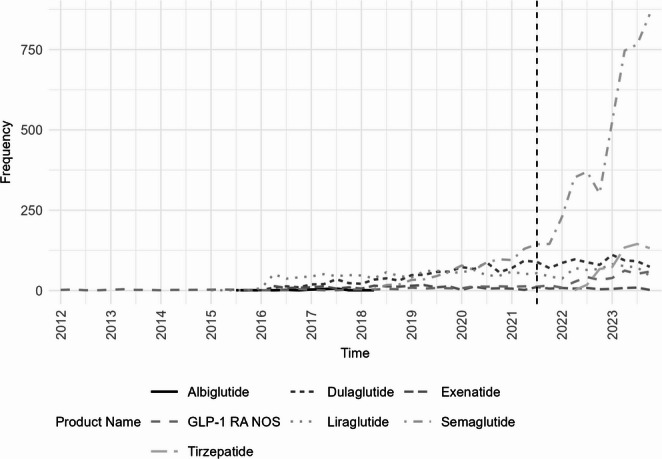
Quarterly counts of GLP-1 receptor agonist exposures stratified by agent; vertical dotted line represents before and after July 1, 2021; GLP-1 RA NOS = not otherwise specified

Before July 2021, exposures most frequently involved liraglutide (33.9%), dulaglutide (29.5%), semaglutide (24.6%), exenatide (6.6%), and GLP-1 RAs not otherwise specified (4.9%), with albiglutide rarely reported (0.5%). After July 2021, semaglutide accounted for 64.2% of cases, followed by dulaglutide (12.7%), liraglutide (8.8%), tirzepatide (8.2%), GLP-1 RAs not otherwise specified (5.1%), and exenatide (1.0%). Non-semaglutide products showed only a slight increase in quarterly call frequency (IRR = 1.02 [95% CI: 1.00, 1.03], *p* = 0.041). These shifts in product distribution over time are shown in Fig. 1.

### Demographics and Exposure Scenario

**Fig. 2:**
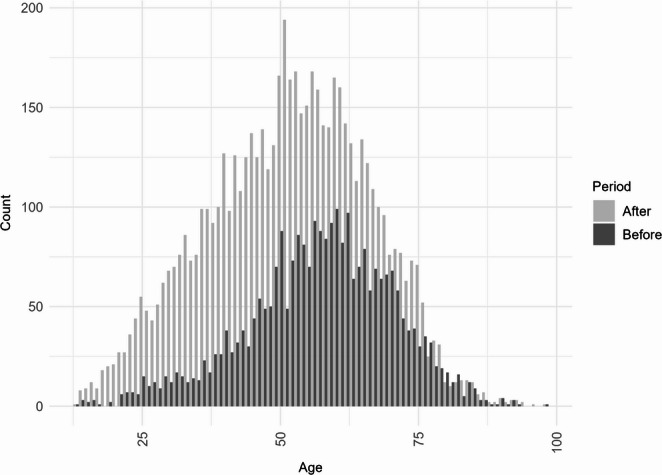
Age distribution (unadjusted) of NPDS calls involving GLP-1 receptor agonists before and after July 1, 2021

The mean age of patients decreased significantly after July 1, 2021 (57.0 years before vs. 51.6 years after; Welch’s t-test, *p* < 0.001), with a Wilcoxon rank-sum test confirming a median difference of 5 years (*p* < 0.001). This age redistribution across categories was likewise indicated by Fisher’s exact test (OR = 0.51 [95% CI: 0.46, 0.56], *p* < 0.001), reflecting a shift toward younger individuals in the post-approval period. When accounting for expected continuation of the pre-approval population, the mean age of new exposures after July 1, 2021 was even lower at 47.5 years, representing a 10-year shift from the pre-approval population (*p* < 0.001). The unadjusted age distributions are shown in Fig. 2. Gender distribution also shifted significantly, with the proportion of females increasing from 68.9% to 78.2% (OR = 1.62 [95% CI: 1.47, 1.78], *p* < 0.001).

Most exposures in both periods occurred at the patient’s own residence (97.4% before vs. 97.5% after). This stability was reflected in the lack of a significant change in the risk of an exposure occurring at a private residence (RR = 1.01 [95% CI: 0.99, 1.03], *p* = 0.508). Note that exposure site (where the exposure occurred) differs from caller site (location at the time of the call). In contrast, chronicity patterns showed a marked shift toward new use: the proportion of exposures coded as acute increased by 11.5% points (47.2% before vs. 58.7% after). This increase coincided with a 37% decrease in the odds of an exposure being classified as acute-on-chronic (45.7% before vs. 34.6% after; OR = 0.63 [95% CI: 0.58, 0.68], *p* < 0.001). Table 1 provides a summary of demographic characteristics across both periods.

**Table 1:** Patient demographics and exposure characteristics before and after July 1, 2021

Characteristic	Category	Before	After
Age (years), mean ± SD	Unadjusted	57.0 ± 13.3	51.6 ± 14.7
	Adjusted^1^	57.0 ± 13.3	47.5
Gender, *n* (%)	Female	2144 (68.9)	5412 (78.2)
	Male	961 (30.9)	1499 (21.7)
	Unknown	8 (0.3)	9 (0.1)
Exposure site, *n* (%)	Own residence	3032 (97.4)	6745 (97.5)
	Other residence	40 (1.3)	78 (1.1)
	Health care facility	7 (0.2)	30 (0.4)
	Workplace	7 (0.2)	13 (0.2)
	Other	21 (0.7)	32 (0.5)
	Public area	1 (0.0)	3 (0.0)
	School	1 (0.0)	2 (0.0)
	Unknown	4 (0.1)	17 (0.2)
Acuity (chronicity), *n* (%)	Acute	1470 (47.2)	4059 (58.7)
	Acute-on-chronic	1422 (45.7)	2392 (34.6)
	Chronic	212 (6.8)	427 (6.2)
	Unknown	9 (0.3)	42 (0.6)

^1^Adjusted values estimate the emergent weight-loss population by subtracting the baseline pre-approval age distribution from the post-approval counts

Exposure reasons were predominantly unintentional therapeutic errors, though their proportion decreased from 85.6% before to 81.0% after approval (RR = 0.95 [95% CI: 0.93, 0.96], *p* < 0.001). Adverse drug reactions were the next most common reason, increasing from 6.3% to 9.6% (RR = 1.52 [95% CI: 1.33, 1.75], *p* < 0.001). Other categories such as intentional misuse, abuse, and unintentional non-therapeutic exposures each comprised less than 5% of cases in both periods. Within therapeutic errors, several specific scenarios showed notable changes: ‘wrong drug’ increased by 4.9% points (95% CI: 3.9, 5.9), ‘wrong time’ increased by 4.9% points (95% CI: 3.5, 6.3), and ‘wrong dose’ decreased by 4.1% points (95% CI: − 6.2, − 2.0). The most frequently documented scenarios by raw count remained ‘wrong dose’ (*n* = 5,171) and ‘wrong time’ (*n* = 1,160).

### Patient Flow, Hospital Flow, and Clinical Outcomes

Patient flow and outcomes changed markedly from the pre-approval period to July 2021 onward. Calls originating from HCFs increased from 15.3% before to 23.6% after approval, representing a 54% increase in the risk of a call originating from a health care setting (RR = 1.54 [95% CI: 1.41, 1.70], *p* < 0.001). Simultaneously, calls from residences declined from 73.7% to 67.2% (RR = 0.91 [95% CI: 0.89, 0.94], *p* < 0.001). Although on-site management remained the most common disposition, its frequency fell by 10.2% points (75.2% to 65.0%), reflecting a 13.6% reduction in the likelihood of being managed without referral (RR = 0.86 [95% CI: 0.84, 0.88], *p* < 0.001). Consequently, the proportion of patients already in, or referred to, a health care facility increased from 23.0% to 33.5%. For this primary outcome, a one-sided test confirmed higher post-approval utilization (*p* < 0.001), with a risk difference of 10.6% points (95% CI: 8.7, 12.4) and a risk ratio of 1.46 (95% CI: 1.36, 1.57).

Medical outcomes also shifted after approval. “No effect” outcomes decreased by 11.6% points (95% CI: − 12.9, − 10.4; *p* < 0.001), while “minor effects” increased by 8.6% points (95% CI: 5.4, 11.7; *p* < 0.001). Moderate effects also became more common (RR = 1.36 [95% CI: 1.14, 1.61], *p* < 0.001), as did cases unable to be followed (RR = 1.22 [95% CI: 1.02, 1.46], *p* = 0.03). Clinical effect duration showed a higher likelihood of symptoms lasting more than 8 h (RR = 2.71 [95% CI: 2.01, 3.64], *p* < 0.001). A detailed summary of patient flow and outcomes is presented in Table 2.

**Table 2:** Patient flow and outcomes before and after July 1, 2021, including caller site, management site, level of health care facility (HCF) care, medical outcomes, and clinical effect duration

Characteristic	Category	Before *n* (%)	After *n* (%)
Caller site	Own residence	2295 (73.7)	4653 (67.2)
	Other residence	44 (1.4)	73 (1.1)
	Workplace	21 (0.7)	38 (0.5)
	Health care facility	476 (15.3)	1635 (23.6)
	Public area	6 (0.2)	11 (0.2)
	School	1 (0.03)	1 (0.01)
	Other	268 (8.6)	500 (7.2)
	Unknown	2 (0.06)	9 (0.1)
Management site	Managed on site (non–HCF)	2341 (75.2)	4498 (65.0)
	Patient already in/en route to HCF	473 (15.2)	1650 (23.8)
	Patient referred to HCF	242 (7.8)	671 (9.7)
	Other	28 (0.9)	48 (0.7)
	Unknown	29 (0.9)	53 (0.8)
Level of HCF care	No HCF care needed	2398 (77.0)	4599 (66.5)
	Treated/evaluated and released	405 (13.0)	1458 (21.1)
	Admitted to noncritical care unit	106 (3.4)	237 (3.4)
	Admitted to critical care unit	13 (0.4)	49 (0.7)
	Lost to follow-up	191 (6.1)	577 (8.3)
Medical outcome	No effect	815 (26.2)	1008 (14.6)
	Minor effect	1978 (63.5)	4988 (72.1)
	Moderate effect	161 (5.2)	486 (7.0)
	Major effect	6 (0.2)	22 (0.3)
	Unable to follow, judged toxic	153 (4.9)	416 (6.0)
Clinical effect duration	≤ 2 h	86 (2.8)	136 (2.0)
	> 2–8 h	117 (3.8)	346 (5.0)
	> 8–24 h	175 (5.6)	572 (8.3)
	> 24 h–3 days	50 (1.6)	301 (4.3)
	> 3 days–1 week	32 (1.0)	193 (2.8)
	> 1 week–1 month	9 (0.3)	30 (0.4)
	> 1 month	1 (0.03)	0 (0.0)
	Anticipated permanent	1 (0.03)	0 (0.0)
	Unknown	106 (3.4)	476 (6.9)
	Not reported	2536 (81.5)	4866 (70.3)

**Fig. 3:**
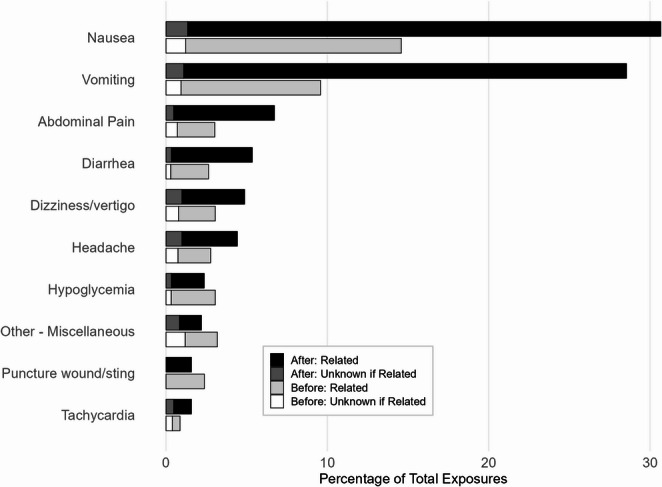
Top ten clinical effects coded as “related” or “unknown if related” to GLP-1 receptor agonist exposures before and after July 1, 2021

**Fig. 4:**
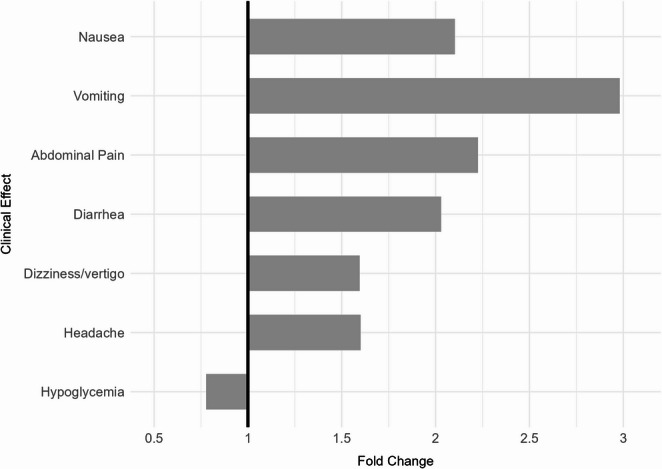
Fold change in the proportion of GLP-1 receptor agonist–related clinical effects after July 1, 2021

The most frequently reported clinical effects were gastrointestinal and neurologic in nature, with clear increases in reports occurring on or after July 1, 2021. These data are presented descriptively to characterize the clinical profile of exposures, rather than to test effect-specific differences between periods. Nausea increased from 14.6% before to 30.7% after. Vomiting increased from 9.6% before to 28.5% after. Abdominal pain increased from 3.0% before to 6.7% after. Diarrhea increased from 2.6% before to 5.3% after. Dizziness or vertigo increased from 3.1% before to 4.9% after. Headache increased from 2.8% before to 4.4% after. Other commonly reported effects increased less or decreased, including hypoglycemia (3.1% before vs. 2.4% after), tachycardia (0.9% before vs. 1.6% after), and hypertension (0.4% before vs. 0.8% after). These distributions are illustrated in Fig. 3, which compares the ten most frequently reported effects before and after approval, and Fig. 4, which presents fold-changes to highlight relative differences between periods.

A sensitivity analysis was performed to evaluate potential bias from grouping symptoms coded as “unknown if related” with those coded as “related.” For the four most common gastrointestinal effects (nausea, vomiting, abdominal pain, and diarrhea), the likelihood of a symptom being coded as “unknown” was significantly lower in the post-approval period (ORs ranging from 0.63 to 0.76; all *p* < 0.001), suggesting improved certainty in symptom attribution over time. Coding specificity remained stable or improved for 9 of the 10 most common effects; the only exception was the broad “other” category, which showed an increase in equivocal coding.

Therapies provided or recommended also increased. The proportion of cases in which antiemetics were recommended rose from 3.6% before to 11.0% after approval (RR = 3.06 [95% CI: 2.56, 3.67], *p* < 0.001). Consistent with increased health care facility utilization, recommendations for intravenous fluids increased from 2.4% to 8.5% (RR = 3.45 [95% CI: 2.80, 4.26], *p* < 0.001).

### Statistical Model

**Fig. 5:**
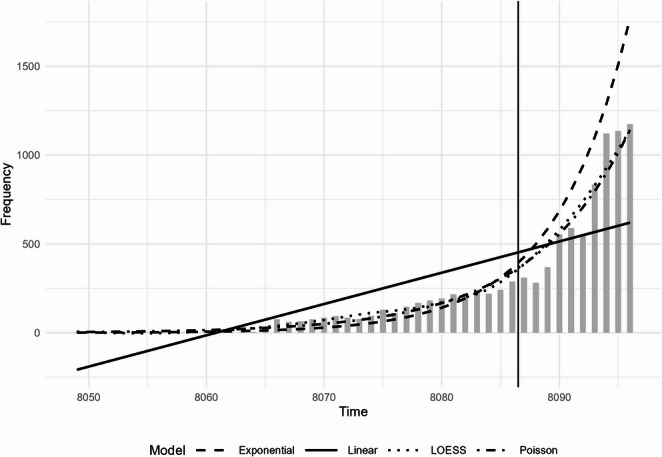
Frequency of NPDS calls related to GLP-1 receptor agonist exposures over time (2012–2023); vertical solid line represents before and after July 1, 2021; quarterly call counts (shaded bars) are overlaid with linear, quadratic, cubic, and quartic models

**Fig. 6:**
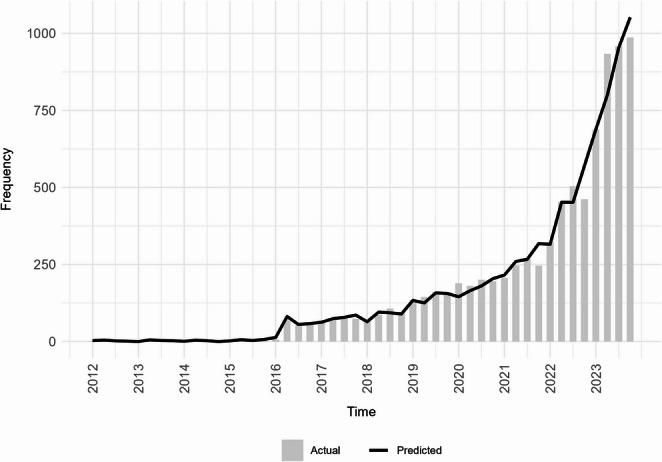
Observed versus Poisson-predicted call frequency by quarter (GLP-1 RA exposures, 2012–2023)

The trend in quarterly call volume showed a significant and non-linear increase over the study period, with a sharp acceleration after mid-2021. To visualize this trajectory, we first explored several polynomial regression models (Fig. 5). Although the cubic and quartic specifications showed close visual agreement with the observed data (*R*^2^=0.97 and 0.99, respectively), these models were used only for descriptive assessment of functional form. For formal inference appropriate to count data, Poisson and negative binomial models were then fit. The Poisson model was preferred for its theoretical suitability and superior overall fit (Fig. 6), confirming that the rapid increase in call volume after the 2021 FDA approval represented a significant change in reporting trajectory. Penalized regression methods were used to assess model parsimony and validate predictor selection.

## Discussion

Overall, the post-approval data describes a younger, predominantly female population experiencing acute therapeutic errors involving semaglutide, accompanied by an increased likelihood of seeking emergency care for their symptoms. Findings from the segmented Poisson model support a significant inflection in the reporting trajectory after the 2021 approval, with semaglutide exposures increasing nearly 10% per quarter compared with only a 1.5% quarterly increase among all other GLP-1 RAs. This divergence is consistent with a reporting inflection temporally aligned with the new weight-management indication and its rapid clinical adoption, rather than background growth in the drug class. The proportion of exposures requiring care in or referral to a health care facility increased from 23.0% before approval to 33.5% after approval (RR = 1.46 [95% CI: 1.36, 1.57], *p* < 0.001), indicating higher health care utilization despite the predominance of mild gastrointestinal effects. Several factors may contribute to this trend. One hypothesis involves patient behavior in the expanding weight-loss population; brisk public demand and rapid clinical adoption could plausibly lead to premature dose escalation or simultaneous use of multiple products (“dose stacking”) [26], although NPDS data cannot confirm this mechanism.

However, this pattern is unlikely to be explained by a single mechanism. Although therapeutic errors remained the predominant exposure circumstance, their proportion decreased slightly, which suggests an evolving exposure profile in which adverse drug reactions and anxiety-related concerns may account for a growing share of the overall volume. The post-approval period was also marked by substantial drug shortages, which forced many patients across both weight-loss and diabetes indications to navigate supply disruptions. These conditions often required switching between formulations, using compounded products with non-standard or unknown concentrations [27, 28], or rationing doses, any of which could increase the likelihood of error independent of patient intent [29]. Patients prescribed GLP-1 RAs for diabetes may be particularly vulnerable to adverse outcomes during such disruptions, given their concurrent use of other glucose-lowering agents and their intrinsic risk of hypoglycemia. Because NPDS does not reliably capture quantitative dosing, product strength, patient weight, or clinical indication, we could not determine which subgroup or mechanism contributed most to the increase in health care utilization.

Additionally, although greater emergency department utilization could suggest increasing case severity, several findings indicate that changes in patient and public reporting behavior likely played a role. Early clinical trials reported that 5–10% of patients experienced mild symptoms of depression or anxiety [10], which may lower the threshold for seeking urgent care for otherwise manageable gastrointestinal or neurologic complaints. Intense media attention and rapid cultural adoption of GLP-1 RAs may have further reduced the threshold for contacting a poison center, contributing modestly to the rise in call volume independent of clinical severity. On the other hand, some patients may also be unaware that poison centers routinely provide guidance for adverse drug events, resulting in direct emergency department presentation rather than initial poison center consultation. Standard management of these adverse effects typically involves symptomatic treatment for acute presentations, such as antiemetics and intravenous fluids, followed by patient-directed non-pharmacologic strategies (smaller meals, slower eating, increased meal frequency) and prescriber-directed pharmacologic adjustments (slower titration, temporary interruption, dose reduction) [29]. Taken together, these behavioral and system-level factors support the conclusion that nondiscretionary utilization of emergency health care resources increased substantially after FDA approval.

The clinical profile of these exposures remained aligned with the expected pharmacologic effects of GLP-1 RAs, with gastrointestinal and neurological symptoms predominating. Nausea and vomiting were the most frequently reported effects, followed by abdominal pain, dizziness, and headache. To evaluate the reliability of these symptom patterns, we examined the certainty of attribution coding. The proportion of symptoms classified as “unknown if related” decreased in the post-approval period. This finding should be interpreted cautiously; increased exposure frequency may have contributed to more consistent symptom attribution, although changes in reporting behavior or underlying case severity cannot be excluded. Nevertheless, the decline in equivocal attribution supports the conclusion that the post-approval rise in reported effects likely reflects true clinical effects rather than misclassification or declining data quality.

Most adverse clinical effects were not serious. However, one death was reported. The patient had a history of obesity and had recently undergone liposuction, with tirzepatide initiated three weeks post-procedure. Ten days later, the patient presented to the emergency department with acute abdominal pain and abdominal distention after several days of constipation. Exploratory laparoscopy revealed a markedly distended colon from rectum to cecum with three areas of ischemia, and surgical resection of the sigmoid, left colon, and splenic flexure was performed. Postoperatively, the patient remained hemodynamically unstable, and nasogastric tube output was concerning for gastrointestinal bleeding. The clinical course progressed to ventricular tachycardia followed by asystole, and resuscitation efforts were unsuccessful. Causality cannot be determined from this single report, but the temporal sequence raises important clinical questions regarding the safety of GLP-1 RA use in the perioperative setting [30].

No cases of pancreatitis were identified in this dataset, as evidenced by the absence of documented elevations in lipase or amylase. However, poison center records and follow-up protocols do not routinely capture laboratory results, limiting the ability to confirm or exclude this diagnosis with certainty. Even so, the lack of reported pancreatitis among more than 10,000 GLP-1 RA exposures suggests that acute pancreatitis related to short-term therapeutic misadventure is uncommon. This observation aligns with prior studies demonstrating inconsistent associations between GLP-1 RAs and pancreatitis risk. The true incidence of pancreatitis or pancreatic cancer from chronic therapeutic use, however, remains unresolved.

Our findings are broadly consistent with prior single-center studies describing GLP-1 RA exposures. In those reports, most cases stemmed from therapeutic errors, and patients typically experienced no symptoms or only mild effects such as nausea, vomiting, abdominal pain, or weakness, with the majority managed safely at home without hospitalization [23, 24]. Hypoglycemia was uncommon, although sporadic cases were noted. Direct comparison across studies should be interpreted cautiously, as poison center follow-up practices and case ascertainment vary across institutions. The present national dataset corroborates these observations at scale and supports the conclusion that isolated GLP-1 RA exposures rarely result in serious toxicity. Because medication reconciliation is incompletely captured in NPDS data, we cannot fully exclude contributions from concomitant agents such as insulin or sulfonylureas, although the lower proportion of diabetic patients in the post-approval cohort likely contributed to the low overall incidence of hypoglycemia. These findings reinforce existing guidance that GLP-1 RAs have a low intrinsic risk of hypoglycemia but may pose increased risk when combined with agents such as insulin or sulfonylureas.

Beyond the specific findings for GLP-1 RAs, this study illustrates the utility of poison center data for near real-time pharmacosurveillance. Raw call volumes are influenced by multiple external factors, including prescribing trends and media attention, which can make it difficult to distinguish true safety signals from background variability. The modeling framework used here provides a formal method for detecting inflection points in reporting trajectories while accounting for underlying long-term growth. This offers a rapid and scalable approach for monitoring post-marketing signals following events such as new FDA approvals, supply disruptions, or shifts in clinical practice. Importantly, the descriptive trends were complemented rather than replaced by the statistical modeling. The regression analyses served to test whether observed increases exceeded what would be expected from prescribing patterns alone, thereby strengthening interpretability while acknowledging that poison center data cannot establish causality.

### Limitations

This study is subject to several limitations inherent to the retrospective analysis of NPDS data. First, reporting to poison centers is voluntary; therefore, these data likely underestimate the true incidence of GLP-1 RA exposures, particularly those managed without consultation or those presenting directly to emergency departments without poison center involvement. Unlike hospital electronic medical records which document confirmed diagnoses and comprehensive treatment courses, poison center records are generated primarily for acute triage and management recommendations. Consequently, data elements rely heavily on self-reporting by callers, which may be subject to recall bias or inaccuracy, and clinical outcomes are often determined at the time of the last contact rather than hospital discharge. Furthermore, these trends must be interpreted in the context of reporting behavior. While recent analyses suggest that ‘stimulated reporting’ (i.e., the Weber effect) may be less prevalent in modern pharmacovigilance where reporting typically tracks dispensing volume [31–34], the unique public profile of GLP-1 RAs warrants a cautious interpretation. Given the intense media coverage and widespread social media attention surrounding these agents, psychosocial factors may have lowered the threshold for contacting a poison center, potentially amplifying call volume independent of true clinical incidence. As such, the observed rise in call volume likely reflects a combination of rapid expansion in the user base and variable degrees of stimulated reporting.

Second, the primary function of a poison center is the immediate assessment and stabilization of the patient, rather than longitudinal data collection. As a result, granular data points such as patient weight, specific medication dosages, and precise clinical indications are frequently missing or incomplete. This precluded a robust dose–response analysis or weight-based risk assessment. We considered using product brand names as a proxy for clinical indication (weight loss vs. diabetes management); however, we rejected this approach due to the widespread off-label prescribing of diabetes formulations for weight loss during periods of drug shortage, which would render such a distinction unreliable.

Additionally, reporting of medical history is limited. Because poison center records do not routinely capture a comprehensive medication reconciliation, we could not reliably control for concomitant medications that may have contributed to the observed clinical effects. This is particularly relevant because key outcomes, especially gastrointestinal symptoms, are nonspecific and overlap with adverse effects from many commonly used drugs. Without a complete list of home medications, we cannot definitively exclude other etiologies or drug-drug interactions. Similarly, we could not adjust for underlying gastrointestinal comorbidities.

Finally, to facilitate statistical modeling, several categorical variables were collapsed into broader groupings, which, while necessary for analysis, may result in a loss of specific clinical detail. Although this approach was necessary for model stability, it reduced the granularity of some clinical categories and may have obscured rare but clinically important distinctions.

### Conclusions

Reports of GLP-1 RA exposures increased substantially after the 2021 approval for weight-loss indications, with semaglutide accounting for most of the post-approval growth. Although the majority of exposures produced mild and self-limited gastrointestinal symptoms, the period was also marked by a significant rise in healthcare facility referrals, increasing from 23.0% before approval to 33.5% after approval. This pattern indicates that even clinically non-severe exposures can generate considerable healthcare utilization. The findings also demonstrate the value of poison center data as a tool for near real-time assessment of emerging post-marketing drug trends. Improved counseling for both patients and prescribers regarding dose titration, product switching, and management of expected gastrointestinal effects may help reduce preventable errors and lessen the resulting burden on emergency services.

## Supplementary Information

Below is the link to the electronic supplementary material.

Supplementary Material 1 (XLSX 3.12 MB)

Supplementary Material 2 (DOCX 341 KB)

Supplementary Material 3 (XLSX 13.3 MB)

Supplementary Material 4 (RMD 56.4 KB)

Supplementary Material 5 (RMD 8.00 KB)

Supplementary Material 6 (RMD 30.1 KB)

Supplementary Material 7 (RMD 22.6 KB)

## Data Availability

America’s Poison Centers (APC) maintains the National Poison Data System (NPDS), which houses de-identified case records of self-reported information collected from callers during exposure management and poison information calls managed by the country’s poison centers. NPDS data do not reflect the entire universe of exposures to a particular substance as additional exposures may go unreported; accordingly, NPDS data should not be construed to represent the complete incidence of U.S. exposures to any substance(s). Exposures do not necessarily represent a poisoning or overdose, and APC is not able to completely verify the accuracy of every report. Findings based on NPDS data do not necessarily reflect the opinions of APC. The de-identified dataset used for this analysis, including both pre- and post-processing spreadsheets, annotated R scripts (markdown files), and supplemental material will be made publicly available at DOI: [10.5281/zenodo.17259427].

## Declarations

### Previous Presentation

Data in this manuscript were previously presented at the American College of Medical Toxicology’s (ACMT) Annual Scientific Meeting, Vancouver, BC, 2025.

## References

[1] Knop FK, Brønden A, Vilsbøll T. Exenatide: pharmacokinetics, clinical use, and future directions. Expert Opin Pharmacother. 2017;18(6):555–71. 10.1080/14656566.2017.1282463. 28085521 10.1080/14656566.2017.1282463

[2] Buse JB, Henry RR, Han J, Kim DD, Fineman MS, Baron AD. Effects of exenatide (exendin-4) on glycemic control over 30 weeks in sulfonylurea-treated patients with type 2 diabetes. Diabetes Care. 2004;27(11):2628–35. 10.2337/diacare.27.11.2628. 15504997 10.2337/diacare.27.11.2628

[3] Kendall DM, Riddle MC, Rosenstock J, Zhuang D, Kim DD, Fineman MS, et al. Effects of exenatide (exendin-4) on glycemic control over 30 weeks in patients with type 2 diabetes treated with metformin and a sulfonylurea. Diabetes Care. 2005;28(5):1083–91. 10.2337/diacare.28.5.1083. 15855571 10.2337/diacare.28.5.1083

[4] DeFronzo RA, Ratner RE, Han J, Kim DD, Fineman MS, Baron AD. Effects of exenatide (exendin-4) on glycemic control and weight over 30 weeks in metformin-treated patients with type 2 diabetes. Diabetes Care. 2005;28(5):1092–100. 10.2337/diacare.28.5.1092. 15855572 10.2337/diacare.28.5.1092

[5] Dushay J, Gao C, Gopalakrishnan GS, Crawley M, Mitten EK, Wilker E, et al. Short-term exenatide treatment leads to significant weight loss in a subset of obese women without diabetes. Diabetes Care. 2012;35(1):4–11. 10.2337/dc11-0931. 22040840 10.2337/dc11-0931 PMC3241299

[6] Buse JB, Rosenstock J, Sesti G, Schmidt WE, Montanya E, Brett JH, et al. Liraglutide once a day versus exenatide twice a day for type 2 diabetes: a 26-week randomised, parallel-group, multinational, open-label trial (LEAD-6). Lancet. 2009;374(9683):39–47. 10.1016/S0140-6736(09)60659-0. 19515413 10.1016/S0140-6736(09)60659-0

[7] Davies MJ, Bergenstal R, Bode B, Kushner RF, Lewin A, Skjøth TV, et al. Efficacy of liraglutide for weight loss among patients with type 2 diabetes: the SCALE diabetes randomized clinical trial. JAMA. 2015;314(7):687–99. 10.1001/jama.2015.9676. 26284720 10.1001/jama.2015.9676

[8] Saxenda (liraglutide) [package insert]. Novo Nordisk Inc.; Plainsboro: 2014.

[9] Miles KE, Kerr JL. Semaglutide for the treatment of type 2 diabetes mellitus. J Pharm Technol. 2018;34(6):281–9. 10.1177/8755122518790925. 34861016 10.1177/8755122518790925 PMC6231279

[10] Wilding JPH, Batterham RL, Calanna S, Davies M, Van Gaal LF, Lingvay I, et al. Once-weekly semaglutide in adults with overweight or obesity. N Engl J Med. 2021;384(11):989–1002. 10.1056/NEJMoa2032183. 33567185 10.1056/NEJMoa2032183

[11] Wegovy (semaglutide) [package insert]. Novo Nordisk Inc.; Plainsboro: 2021.

[12] Li P, Varghese JS, Shah MK, Galindo RJ, Pasquel FJ, Ali MK, et al. Prescribing trends of glucagon-like peptide 1 receptor agonists for type 2 diabetes or obesity. JAMA Netw Open. 2025;8(10):e2540890. 10.1001/jamanetworkopen.2025.40890. 41171277 10.1001/jamanetworkopen.2025.40890 PMC12579341

[13] Drucker DJ. GLP-1 physiology informs the pharmacotherapy of obesity. Mol Metab. 2022;57:101351. 10.1016/j.molmet.2021.101351. 34626851 10.1016/j.molmet.2021.101351 PMC8859548

[14] Nauck MA, Quast DR, Wefers J, Meier JJ. GLP-1 receptor agonists in the treatment of type 2 diabetes - state-of-the-art. Mol Metab. 2021;46:101102. 10.1016/j.molmet.2020.101102. 33068776 10.1016/j.molmet.2020.101102 PMC8085572

[15] Klausen MK, Thomsen M, Wortwein G, Fink-Jensen A. The role of glucagon-like peptide 1 (GLP-1) in addictive disorders. Br J Pharmacol. 2022;179(4):625–41. 10.1111/bph.15677. 34532853 10.1111/bph.15677 PMC8820218

[16] Elashoff M, Matveyenko AV, Gier B, Elashoff R, Butler PC. Pancreatitis, pancreatic, and thyroid cancer with glucagon-like peptide-1–based therapies. Gastroenterology. 2011;141(1):150–6. 10.1053/j.gastro.2011.02.018. 21334333 10.1053/j.gastro.2011.02.018 PMC4404515

[17] Sodhi M, Rezaeianzadeh R, Kezouh A, Etminan M. Risk of gastrointestinal adverse events associated with Glucagon-Like Peptide-1 receptor agonists for weight loss. JAMA. 2023;330(18):1795–7. 10.1001/jama.2023.19574. 37796527 10.1001/jama.2023.19574 PMC10557026

[18] Dankner R, Murad H, Agay N, Olmer L, Freedman LS. Glucagon-Like Peptide-1 receptor agonists and pancreatic cancer risk in patients with type 2 diabetes. JAMA Netw Open. 2024;7(1):e2350408. 10.1001/jamanetworkopen.2023.50408. 38175642 10.1001/jamanetworkopen.2023.50408 PMC10767614

[19] Cao C, Yang S, Zhou Z. GLP-1 receptor agonists and pancreatic safety concerns in type 2 diabetic patients: data from cardiovascular outcome trials. Endocrine. 2020;68(3):518–25. 10.1007/s12020-020-02223-6. 32103407 10.1007/s12020-020-02223-6

[20] Ja’arah D, Al Zoubi MS, Abdelhady G, Rabi F, Tambuwala MM. Role of glucagon-like peptide-1 (GLP-1) receptor agonists in hypoglycemia. Clin Med Insights Endocrinol Diabetes. 2021;14:11795514211051697. 10.1177/11795514211051697. 34690504 10.1177/11795514211051697 PMC8527576

[21] Zhao Z, Tang Y, Hu Y, Zhu H, Chen X, Zhao B. Hypoglycemia following the use of glucagon-like peptide-1 receptor agonists: a real-world analysis of post-marketing surveillance data. Ann Transl Med. 2021;9(18):1482. 10.21037/atm-21-4162. 34734034 10.21037/atm-21-4162 PMC8506728

[22] Stauder U, Enginee D, Elton H, Penfornis A, Edelman S. Comparative assessment of Lixisenatide, Exenatide, and liraglutide pen devices: a pilot user-based study. J Diabetes Sci Technol. 2014;8(1):123–31. 10.1177/1932296813511733. 24876548 10.1177/1932296813511733 PMC4454110

[23] Muschler K, Muschalek R, Hoyte C. Characterization of glucagon-like peptide-1 (GLP-1) agonist exposures reported to a single united States poison center. Clin Toxicol Phila Pa. 2025;63(2):133–6. 10.1080/15563650.2024.2444642. 10.1080/15563650.2024.2444642 39803696

[24] Marshall S, Ryan E, Rivera J, Reynolds L, Atti S. GLP-1 receptor agonist exposures are increasingly common and generally associated with mild symptoms: a single poison center experience. J Med Toxicol. 2024;20(3):278–85. 10.1007/s13181-024-01008-x. 38861153 10.1007/s13181-024-01008-x PMC11288212

[25] FDA approves game changer semaglutide for weight loss. Medscape. 2021 [cited 2025 November 24]; Available from: https://www.medscape.com/viewarticle/952441.

[26] Chiappini S, Vickers-Smith R, Harris D, Papanti Pelletier GD, Corkery JM, Guirguis A, et al. Is there a risk for semaglutide misuse? Focus on the Food and Drug Administration’s FDA adverse events reporting system (FAERS) pharmacovigilance dataset. Pharmaceuticals. 2023;16(7):994. 10.3390/ph16070994. 37513906 10.3390/ph16070994 PMC10384093

[27] Lambson JE, Flegal SC, Johnson AR. Administration errors of compounded semaglutide reported to a poison control center—case series. J Am Pharm Assoc. 2023;63(5):1643–5. 10.1016/j.japh.2023.06.017. 10.1016/j.japh.2023.06.017 37392810

[28] Wiener BG, Gnirke M, Vassallo S, Smith SW, Su MK. Challenges with glucagon-like peptide-1 (GLP-1) agonist initiation: a case series of semaglutide overdose administration errors. Clin Toxicol Phila Pa. 2024;62(2):131–3. 10.1080/15563650.2024.2322049. 10.1080/15563650.2024.2322049 38470137

[29] Gorgojo-Martínez JJ, Mezquita-Raya P, Carretero-Gómez J, Castro A, Cebrián-Cuenca A, de Torres-Sánchez A, et al. Clinical recommendations to manage gastrointestinal adverse events in patients treated with Glp-1 receptor agonists: a multidisciplinary expert consensus. J Clin Med. 2022;12(1):145. 10.3390/jcm12010145. 36614945 10.3390/jcm12010145 PMC9821052

[30] Kindel TL, Wang AY, Wadhwa A, Schulman AR, Sharaiha RZ, Kroh M, et al. Multisociety clinical practice guidance for the safe use of glucagon-like peptide-1 receptor agonists in the perioperative period. Clin Gastroenterol Hepatol. 2025;23(12):2083–5. 10.1016/j.cgh.2024.10.003. 39480373 10.1016/j.cgh.2024.10.003

[31] Hartnell NR, Wilson JP, Patel NC, Crismon ML. Adverse event reporting with selective serotonin-reuptake inhibitors. Ann Pharmacother. 2003;37(10):1387–91. 10.1345/aph.1C522. 14519041 10.1345/aph.1C522

[32] McAdams MA, Governale LA, Swartz L, Hammad TA, Dal Pan GJ. Identifying patterns of adverse event reporting for four members of the angiotensin II receptor blockers class of drugs: revisiting the Weber effect. Pharmacoepidemiol Drug Saf. 2008;17(9):882–9. 10.1002/pds.1633. 18636418 10.1002/pds.1633

[33] Hoffman KB, Dimbil M, Erdman CB, Tatonetti NP, Overstreet BM. The Weber effect and the united States food and drug administration’s adverse event reporting system (FAERS): analysis of sixty-two drugs approved from 2006 to 2010. Drug Saf. 2014;37(4):283–94. 10.1007/s40264-014-0150-2. 24643967 10.1007/s40264-014-0150-2 PMC3975089

[34] Modgill V, Dormegny L, Lewis DJ. Reporting rates of adverse reactions to specialty care medicines exhibit a direct positive correlation with patient exposure: a lack of evidence for the Weber effect. Br J Clin Pharmacol. 2020;86(12):2393–403. 10.1111/bcp.14342. 32374028 10.1111/bcp.14342 PMC7688531

