# Supplementary material for: National Poison Center Trends in GLP-1 Receptor Agonist Exposures Following FDA Approval for Weight Loss
Source: J Med Toxicol. 2026 Feb 3;22(2):275–85. doi: 10.1007/s13181-026-01121-z (PMC13076849; doi:10.1007/s13181-026-01121-z)
Supplement: Supplementary file 2 — Supplementary Material 2 (DOCX 341 KB) [file 13181_2026_1121_MOESM2_ESM.docx]

**Weighing In: National Poison Center Trends in GLP-1 Receptor Agonist Exposures Following FDA Approval for Weight Loss**

# 1 Supplemental Methods

## 1.1 Data Source and Software Environment

We conducted a retrospective analysis of human exposures to glucagon-like peptide-1 receptor agonists (GLP-1 RAs) reported to the U.S. National Poison Data System^®^ (NPDS^®^) from January 1, 2012, through December 31, 2023. The raw data were provided as a multi-sheet Microsoft Excel workbook, with each worksheet corresponding to a specific data domain (e.g., Demographics, Clinical Effects).

All data cleaning, analysis, and visualization were performed using R version 4.3.2 in the RStudio environment. The following key R packages were used: readxl for data import; tidyverse (including dplyr, tidyr, and ggplot2) for data manipulation and visualization; openxlsx for exporting results; lubridate for date-time processing; MASS and glmnet for statistical modeling; and conflicted to manage function namespaces.

## 1.2 Data Cleaning and Preprocessing

For each worksheet, we removed columns containing only a single unique value, except for variables identified by toxicology consultants as clinically informative pertinent negatives (e.g., pancreatitis and hematemesis). Original data were preserved, and new cleaned data frames were created.

Within the Demographics sheet, we verified uniqueness of case identifiers and standardized variables. Product names were harmonized to generic categories (e.g., Ozempic, Rybelsus, and Wegovy collapsed into semaglutide). A catch-all category (GLP-1 RA NOS) was created for rare or missing products. Age was recoded into categorical ranges by decade, supplemented by the Age Unit field, and consolidated into a single Age Range variable. Implausible or missing Weight values (present in 77.7% of cases) led to exclusion of the weight variable. Level of Health Care Facility Care and Medical Outcome were collapsed into fewer ordered categories.

Within the Clinical Effects sheet, symptom fields were recoded such that missing values were explicitly labeled *Not Reported*. For Routes, multiple binary columns were collapsed into a single categorical variable. For Scenarios, binary indicators were consolidated into five “rights” categories (wrong *patient*, *drug*, *time*, *dose*, *route*), plus Multiple Errors and Unknown. In the Therapy sheet, therapy variables were recoded to distinguish recommended, performed, and non-recommended/non-performed (NR/NP) statuses.

## 1.3 Data Integration and Ordinality

All worksheets were joined by the unique case identifier. Start date and time fields were consolidated into a single datetime variable (Start_DateTime), and quarter-year variables were created for time-series modeling. Redundant or non-informative fields were dropped at this stage. The final preprocessed dataset was saved to an Excel workbook (Joined_Preprocessed_GLP1_Data.xlsx), and a companion file documenting the ordinal variable structure was exported as an RDS object (ordinal_metadata.rds).

Several variables were explicitly treated as ordinal: Age Range, Chronicity, Exposure Duration, Level of HCF Care, Medical Outcome, and Clinical Effect Duration. Symptom fields were uniformly recoded to ordered factors with four levels: *Not Reported*, *Not Related*, *Unknown If Related*, and *Related*. Ordinal encodings were applied using metadata and carried forward to all analyses.

## 1.4 Statistical Analysis

### 1.4.1 Exploratory Visualization

Frequency plots were generated for categorical variables, with bar charts and contingency heatmaps used to assess distributional patterns. For symptom fields, we identified the ten most frequently reported outcomes overall and stratified by pre- and post-July 1, 2021 (the date of FDA approval of once-weekly semaglutide for weight loss). Fold-change calculations were performed to assess changes in symptom reporting between periods.

### 1.4.2 Temporal Modeling of Call Frequency

Counts of GLP-1 RA exposures were aggregated by quarter, half-year, and year. Models included linear, quadratic, exponential, Poisson, negative binomial, and LOESS. Goodness-of-fit was evaluated using R² and residual diagnostics. Poisson and LOESS models provided the best fit; Poisson was preferred for its theoretical appropriateness for count data. Segmented regression compared trends before and after July 2021.

### 1.4.3 Drug-Specific Incidence

Exposures were grouped by product category, and incidence was visualized with combined and faceted line plots. A vertical reference line was added at July 2021 to highlight the approval date. Shares of exposures by product were calculated before and after this threshold.

### 1.4.4 Demographics Analysis

Age was analyzed in both continuous and categorical formats. Mean, median, and standard deviation were calculated for pre- and post-approval groups. To account for persistence of baseline exposure patterns, an adjusted post-approval distribution was created by subtracting pre-approval frequencies at each age from post-approval frequencies. Negative values were truncated at zero to avoid spurious results. Means and standard deviations were recalculated from this adjusted dataset, which was interpreted as approximating “new” post-approval cases distinct from the pre-existing diabetes population. Age was further categorized by decade-based ranges, excluding records labeled “Unknown adult (≥20 years).” Contingency tables compared frequencies of each age range before and after July 2021. Gender distributions were summarized by frequency and percentage across periods.

### 1.4.5 Statistical Testing

Continuous age distributions were compared using Welch’s t-test for mean differences and the Wilcoxon rank-sum test for medians. Fisher’s exact test was applied to binary categorizations of age (younger vs. older than the cohort median). Chi-square tests compared categorical *Age Range* distributions across periods, with expected cell counts examined for validity. Gender distributions were tested for differences in proportions using chi-square.

All demographic analyses were performed twice: once using the full dataset and once using the adjusted post-approval dataset described above. This two-step approach allowed evaluation of whether demographic shifts reflected continuation of baseline exposures or emergence of new patterns following FDA approval for weight loss.

Call frequency was modeled to evaluate temporal patterns and predictors of GLP-1 RA exposures. Data were sanitized prior to modeling by standardizing column names (spaces and special characters replaced with underscores), excluding identifiers and redundant variables, and removing factor variables with fewer than two levels.

Two time variables were constructed: Time Since Start (days since January 1, 2012) and Time Since Approval (days since July 1, 2021, truncated at zero for pre-approval cases). Quarter-year variables were derived using lubridate::floor_date, and call frequencies were aggregated per quarter. Quarters since both reference dates were calculated explicitly to allow trend modeling relative to approval.

### 1.4.6 Regression Models

Case-level data were collapsed into a frequency dataset (newdat) by unique combinations of product, age range, chronicity, level of health care facility care, medical outcome, and time variables. This reduced redundancy and created the count response variable (Frequency). Factors with “Unknown” values were recoded as NA, then converted to numeric orderings for regression. Columns containing only NAs or single-level factors were excluded. Several modeling approaches were fit:

- Linear regression on log-transformed frequency as an initial check. Interaction terms such as semaglutide × time since approval were considered.
- Poisson regression with main effects and selected interactions. A full factorial was attempted, but unstable or collinear terms were dropped (e.g., chronicity × outcome). Predicted vs. observed quarterly frequencies were plotted, and R² was calculated from correlation of observed and predicted totals.
- Negative binomial regression using glm.nb (MASS) to account for overdispersion. Observed and fitted values were compared graphically.
- LASSO and elastic net regularization using glmnet. Cross-validation was applied to select the optimal λ. Nonzero coefficients were extracted, and reduced equations were derived. Models were compared in terms of retained variables and predictive accuracy.
- Stepwise selection (forward and backward) using stepAIC was applied to Poisson specifications to compare parsimony versus fit.

Goodness-of-fit was evaluated by R², AIC, and visual comparison of predicted vs. actual call frequencies by quarter. Plots included predicted vs. observed trajectories over time and scatterplots of observed vs. predicted frequencies with reference lines. Overfitting was monitored by comparing full Poisson models to regularized (LASSO/elastic net) and stepwise-selected models. All models were implemented in R with tidyverse, MASS, mgcv, and glmnet. Figures were generated with ggplot2, patchwork, and gt. Model summaries and regression coefficient tables were exported to Excel and image formats.

After evaluating these models, the Poisson regression model was selected for the final analysis based on its strong performance on goodness-of-fit metrics and its theoretical appropriateness for count data. The suitability of this model is demonstrated in **Supplemental Fig. 1**, where the fitted values closely track the observed quarterly call counts, accurately capturing the sharp acceleration in exposures after the July 2021 FDA approval. The predictor and response variables included in this final model are described in **Supplemental Table 1**.

**Fig. S1**. Observed vs. Fitted Quarterly Call Frequencies from the Final Poisson Regression Model.


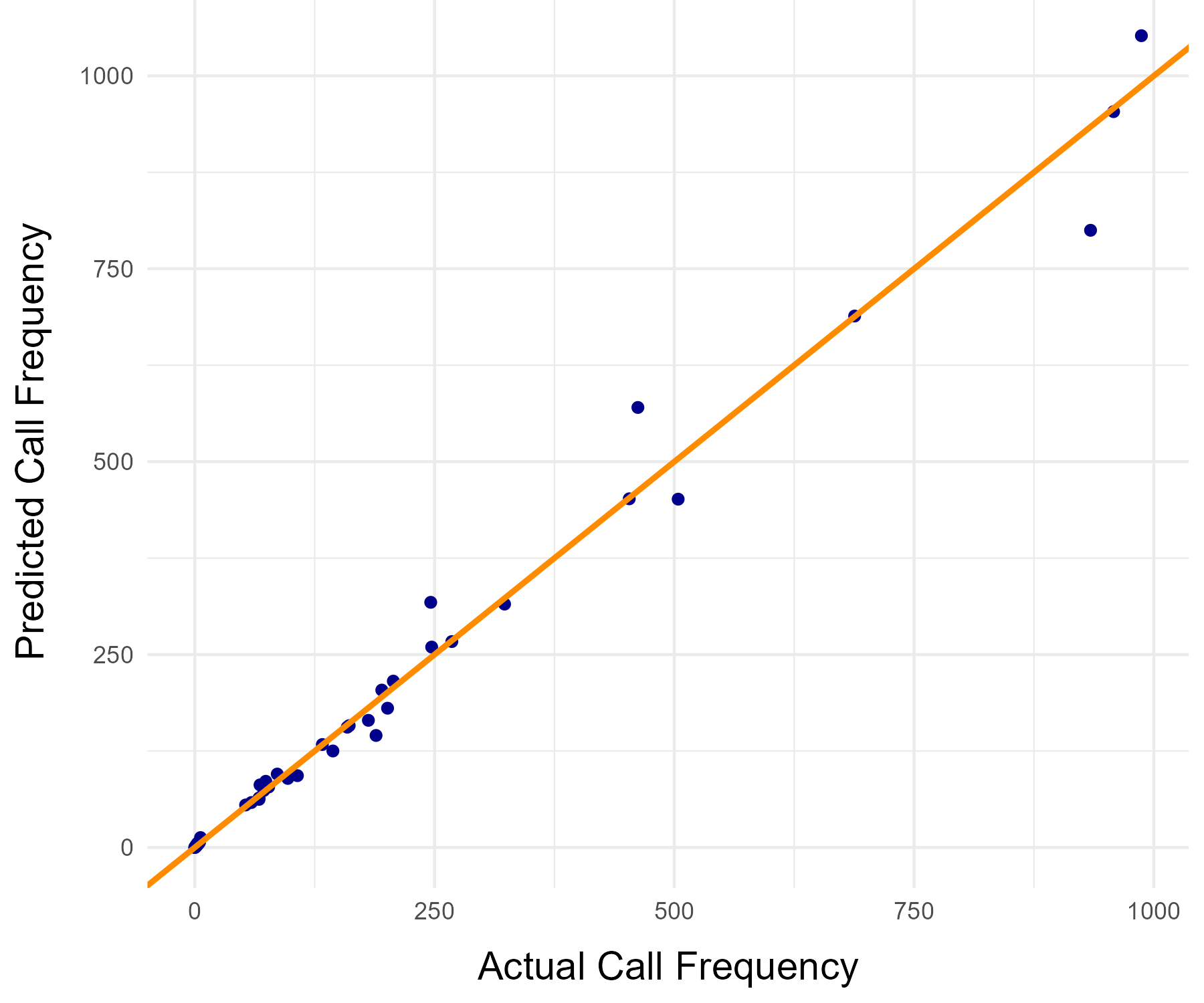


Observed quarterly call counts for GLP-1 RA exposures (orange bars) are overlaid with the fitted values from the final Poisson regression model (blue line). The model closely tracks the observed data, accurately capturing the sharp increase in call volume following the July 1, 2021, FDA approval of once-weekly semaglutide for weight loss (vertical dashed line).

**Table S1**. Description of Variables Used in Temporal Regression Models


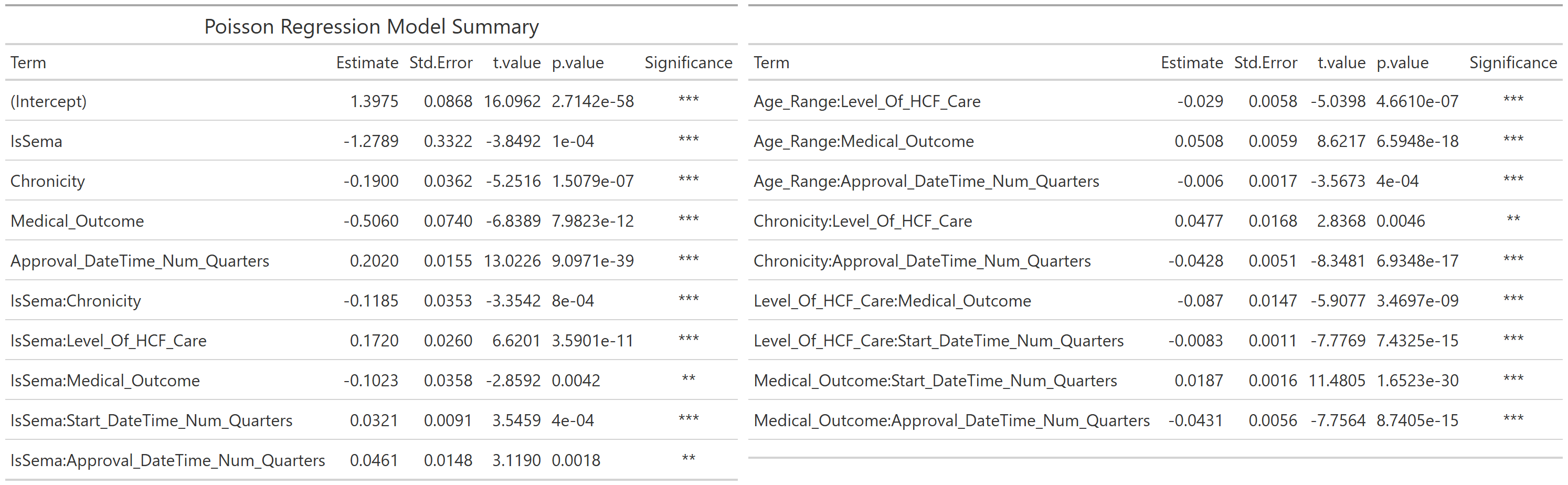


Description, data type, and role of variables included as predictors or as the response in the Poisson regression models. The model's response variable is Frequency, the count of calls per quarter. Time is represented by two variables: Quarter_Since_Start for the overall long-term trend and Quarter_Since_Approval to specifically capture changes after the July 2021 FDA approval. Key categorical predictors such as Medical_Outcome_Ordered and Age_Range_Ordered were treated as ordered factors in the analysis. See Supplemental Table S1 for the coefficient estimates for these variables in the final model.
